# *Lactobacillus salivarius* alleviates inflammation via NF-κB signaling in ETEC K88-induced IPEC-J2 cells

**DOI:** 10.1186/s40104-020-00488-5

**Published:** 2020-08-03

**Authors:** Jiayun Qiao, Zeyang Sun, Dongmei Liang, Haihua Li

**Affiliations:** 1 College of Life Sciences, Tianjin Key Laboratory of Animal and Plant Resistance, Tianjin Normal University, Tianjin, 300387 People’s Republic of China; 2 Tianjin Key Laboratory of Agricultural Animal Breeding and Healthy Husbandry, College of Animal Science and Veterinary Medicine, Tianjin Agricultural University, 22 Jinjing Road, Tianjin, 300384 People’s Republic of China

**Keywords:** ETEC K88, Inflammatory response, IPEC-J2, *L. salivarius*

## Abstract

**Background:**

Enterotoxigenic *Escherichia coli* (ETEC) K88 commonly colonize in the small intestine and keep releasing enterotoxins to impair the intestinal barrier function and trigger inflammatory reaction. Although *Lactobacillus salivarius* (*L. salivarius*) has been reported to enhance intestinal health, it remains to be seen whether there is a functional role of *L. salivarius* in intestinal inflammatory response in intestinal porcine epithelial cell line (IPEC-J2) when stimulated with ETEC K88. In the present study, IPEC-J2 cells were first treated with *L. salivarius* followed by the stimulation of ETEC K88 for distinct time period. ETEC K88 adherent status, pattern recognition receptors (PRRs) mRNA, mitogen-activated protein kinase (MAPK) and nuclear factor-κB (NF-κB) activation, the release of pro-inflammation cytokines and cell integrity were examined.

**Results:**

Aside from an inhibited adhesion of ETEC K88 to IPEC-J2 cells, *L. salivarius* was capable of remarkably attenuating the expression levels of interleukin (IL)-1β, tumor necrosis factor-α (TNF-α), IL-8, Toll-like receptor (TLR) 4, nucleotide-binding oligomerization domain (NOD)-like receptor pyrin domain-containing protein (NLRP) 3 and NLRP6. This alternation was accompanied by a significantly decreased phosphorylation of p38 MAPK and p65 NF-κB during ETEC K88 infection with *L. salivarius* pretreatment. Western blot analysis revealed that *L. salivarius* increased the expression levels of zona occludens 1 (ZO-1) and occludin (*P* < 0.05) in ETEC K88-infected IPEC-J2 cells. Compared with ETEC K88-infected groups, the addition of *L. salivarius* as well as extra inhibitors for MAPKs and NF-κB to ETEC K88-infected IPEC-J2 cells had the capability to reduce pro-inflammatory cytokines.

**Conclusions:**

Collectively, our results suggest that *L. salivarius* might reduce inflammation-related cytokines through attenuating phosphorylation of p38 MAPK and blocking the NF-κB signaling pathways. Besides, *L. salivarius* displayed a potency in the enhancement of IPEC-J2 cell integrity.

## Background

ETEC K88, a common environmental pathogen, has been considered as a major cause of diarrhea in post-weaned piglets, and then contribute to an increased mortality and poor performance during weaning transition [1]. Since the application of certain antibiotics for food-producing animals has been widely restricted, a growing demand for alternative feed additives and ingredients needs to be satisfied in an attempt to improve health condition, boost productive performance and avoid vexing problems like the development of bacterial resistance. Probiotics are tiny living microorganisms which, when consumed by the host as part of food sources with adequate amounts, could confer numerous benefits on the health condition and serve as a potential strategy for certain disease prevention and treatment. A large number of studies have shown that *Lactobacillus,* an extensively used species both in humans and animals [2, 3], is widely known for its capability to improve intestinal health and promote growth performance. This improvement could be obtained by preventing other pathogenic bacteria from attaching to the intestine [4, 5], alleviating existed intestinal damage [6], restoring impaired intestinal barrier [7] and enhancing inadequate immune system [8]. For instance, with reference to a previous knowledge, *L. salivarius* B1 was found to facilitate an early colonization in duodenal mucosa and an up-regulated expression of porcine β-defensin-2 (pBD-2) in saliva might be part responsible for the immune response; this up-regulation was tightly correlated with the administration of successive doses of *L. salivarius* B1 [9]. However, the exact mechanisms concerning *L. salivarius* on health modulation, such as inflammatory response and immunology regulation, have not yet been elucidated.

The intestinal mucosal barrier is an integral part for maintaining the homeostasis of the gut micro-environment. The intestinal epithelial cells are important components of this barrier while the intestinal tight junctions adjacent to individual cells act as another physical structure. This complicated tight junction complex consists of approximately 50 proteins which are basically classified into structural and functional units [10, 11]. The pivotal role of tight junctions is to seal the gap between epithelial cells and thus restrict microorganisms or other antigens from infiltrating into the systematic circulation [12]. Studies have offered perspectives that the treatment of *Lactobacillus* could augment the expression levels of various tight junction proteins, including claudin-1, occludin and ZO-1. Since tight junctions are responsible for the proper permeability, these elevated protein levels have presented an efficacy of probiotics in maintaining the regular environment of pig intestinal mucosa and reducing porcine diarrhea incidence [13, 14].

TLRs and NOD-like receptors (NLRs), defined as two essential groups of membrane receptors of intestinal epithelial cells, play critical roles in the innate immune system by identifying the conserved pathogen-associated molecular patterns (PAMPs) from various invading microbes [15]. It is well understood that this identification could consequently trigger the activation of MAPK, NF-κB and caspase-1 and after which an assortment of inflammation-related cytokines will be released, such as TNF-α, IL-8, and IL-1β. Thus, these signaling pathways are likely to get involved in the host defensive and inflammatory system [16]. One previous research discovered that *L. plantarum* administration could bring not only the inhibition of TLR4-induced MAPK and NF-κB signaling pathways but also the down-regulation of IL-1β, TNF-α and IL-6. The above-mentioned alternations of immunostimulatory cytokines offered a possibility that *L. plantarum* could be considered as a key regulator of the innate immune system in the infected individuals and that symptoms of pathogenic ETEC K88-induced diseases might be relieved with the assistance of this strain [17]. Besides, *Lactobacillus* could also modulate the expression levels of NOD-1 and NOD-2, and the change of which could be likely connected with immunomodulatory activities [18]. However, it remains unclear whether *L. salivarius* could hinder the ETEC K88 infection by mediating cellular immunity via regulating TLRs, NLRs and corresponding downstream targets.

Even though a number of investigators have clarified promising outcomes on the health regulation brought by several *Lactobacillus* strains, the knowledge about the potential influence of *L. salivarius* on the inflammatory response is still ambiguous. In this study, ETEC K88 was selected as a pathogen to infect IPEC-J2 cells and then a variety of parameters were measured with the treatment of *L. salivarius*, including the expression levels of certain molecules involved in the pathogenic recognition, activation of MAPK or NF-κB signaling pathways, the secreted cytokines as well as the cell integrity. Our study aims to explore the protective role of *L. salivarius* in regulating inflammatory response when pathogenic invasion occurs in the intestinal epithelial cells.

## Materials and methods

### The culture of IPEC-J2 cells

IPEC-J2 cells were cultured in 89% Roswell Park Memorial Institute (RPMI) Medium 1640 basic (Gibco, Grand Island, NY, USA) supplemented with 10% fetal bovine serum (Gibco, Grand Island, NY, USA) and 1% penicillin/streptomycin (Solarbio, Beijing, China) at 37°C with 5% CO_2_ in the humidified incubator. After growing to 80–90% confluence, the cultured cells were digested with 0.25% trypsin-EDTA for later passage or protein extractions.

### Bacterial strains

*L. salivarius* was isolated and purified from fresh feces of 28-day-old healthy weaned-piglets. Certain biochemical methods for identifying those isolated *L. salivarius* samples were used, such as the gene sequencing of 16S rRNA and the homology analysis. *L. salivarius* was first incubated in *Lactobacillus* MRS broth at 37 °C for 24 h. After dilution with fresh medium, the cultures were incubated under the same condition until the appearance of logarithmic phase. *L. salivarius* was harvested from the broth at 5,000 r/min for 10 min at 4 °C. The precipitated bacteria and the glycerol were mixed with the ratio of 1:2. Meanwhile, the concentration was confirmed through serial dilutions followed by colony forming unit (CFU) counts on de Man, Rogosa and Sharpe (MRS) agar after 24 h incubation under anaerobic environment. All the cultured bacteria were finally stored at − 80 °C for further performances. The ETEC K88 strains were obtained from the China Veterinary Culture Collection Center (CVCC1502). The performances of bacterial culturing, harvesting, and counting were conducted as previously described [8].

### ETEC K88 adhesion assay

IPEC-J2 cells were inoculated into two 24-well plates (Corning, Inc., Corning City, NY, USA) with a density of 3 × 10^5^ cells/well for 50% confluence. *L. salivarius* (1 × 10^5^ CFU/well) were then supplemented into these wells for 3 h, and after which cells were stimulated with ETEC K88 (1 × 10^3^ CFU/well) for 0, 3, 6 and 24 h, respectively. The applied concentration and stimulation time of both *L. salivarius* and ETEC K88 were designed based on a standard that the normal adherent status of bacteria was not affected while cell monolayer was not destroyed. Pig lactate dehydrogenase (LDH) enzyme-linked immunosorbent assay (ELISA) Kit was utilized to detect whether the cellular structure was damaged. IPEC-J2 cells with different treatments were washed three times with RPMI Medium 1640 basic to remove non-adherent ETEC K88. 100 μL of 0.5% TritonX-100 was then added to each well of the plate to lyse the cells for 8 min in the 37 °C incubator, and 900 μL of phosphate buffer saline (PBS) was used to stop the cell lysis. The lysate was serially diluted and incubated in Luria-Bertani (LB)-Agar dishes for 12 h to quantitate bacterial populations. At this time, the adherent population of ETEC K88 (C1) was the total adherent population of both phagocytes and those ETEC K88 sticking to the surface of IPEC-J2 cells. The other plate was added with 0.4 mL culture medium containing 100 μg/mL gentamycin per well for 2 h to kill IPEC-J2 cells with ETEC K88 attachment on the cellular surface. The gentamicin was finally discarded and the cells were lysed and terminated as described above. In this case, the remained cells in fact were those with engulfed ETEC K88 and the phagocytosis population of ETEC K88 (C2) was correspondingly calculated. ETEC K88 adherent population = C1−C2. The adherent ratio = (C1−C2)/C1 × 100%.

### Quantitative real-time polymerase chain reaction (qPCR)

IPEC-J2 cells were pretreated with *L. salivarius* and followed by ETEC K88 infection as described above. All the treated IPEC-J2 cells were harvested at 3, 6 or 12 h post-infection, respectively, for total RNA extraction by using TRIzol reagent (Takara Biotechnology, Dalian, China). One μg of qualified RNA was used for cDNA synthesis using Moloney Murine Leukemia Virus Reverse Transcriptase (Promega, Madison, WI, USA) to later examine the expression levels of TLR2, TLR4, NLRP3 and NLRP6. The qPCR was performed based on designed primers (Table 1) and the relative expressions of target genes were analyzed as previously described [8]. All samples were run in triplicate.

**Table 1:** Sequences of oligonucleotide primers used for qPCR

Target	Primer	Sequence (5′ to 3′)	Size, bp
TLR2	Forward	TCATCTCCCAAATCTGCGAAT	167
	Reverse	GGCTGATGTTCTGAATTGACCTC	
TLR4	Forward	CCGTCATTAGTGCGTCAGTTCT	100
	Reverse	TTGCAGCCCACAAAAAGCA	
NLRP3	Forward	AGCAGATTCCAGTGCATCAAAG	76
	Reverse	CCTGGTGAAGCGTTTGTTGAG	
NLRP6	Forward	TCAACCGCCTCTTCAGCC	116
	Reverse	CGCCCAGTCGTACAGGATTT	
GAPDH	Forward	GAAGGTCGGAGTGAACGGAT	150
	Reverse	CATGGGTAGAATCATACTGGACA	

### Cellular lysate preparation

IPEC-J2 cells were lysed with protease inhibitor-lysis solution for 10 min on ice, and cell debris were then removed by centrifugation at 12,000 r/min for 15 min at 4 °C. Total protein concentration was determined using the BCA Protein Assay Kit (Applygen Technologies Inc., Beijing, China).

### Western blotting

The extracted proteins from different treated cells were mixed with Laemmli sample buffer for 12% SDS-PAGE and a consistent amount of each protein sample was loaded. After electrophoresis, the gel with separated proteins was released from the plastic case and those protein samples were electrophoretically transferred onto polyvinylidene difluoride (PVDF) membrane (Millipore, Billerica, MA, USA). This membrane with migrated proteins was then coated with blocking buffer for 2 h at room temperature to prevent unspecific bindings and incubated with optimized diluted primary anti-bodies for proteins of interests for 2 h at room temperature, including anti-ZO-1 (Abcam, Cambridge, UK), anti-occludin (Abcam, Cambridge, UK), anti-NF-кB p65 (Cell Signaling Technology, Danvers, MA, USA), anti-phospho-NF-кB p65 (Cell Signaling Technology, Danvers, MA, USA), anti-p38 MAPK (Cell Signaling Technology, Danvers, MA, USA), anti-phospho-p38 MAPK (Cell Signaling Technology, Danvers, MA, USA), anti-ERK MAPK (Cell Signaling Technology, Danvers, MA, USA), anti-phospho-ERK MAPK (Cell Signaling Technology, Danvers, MA, USA) and anti-GAPDH (Abcam, Cambridge, UK) antibodies. After 3 times rinsing with Tris-Buffered-Saline with Tween (TBST), the membrane was incubated with horseradish peroxidase (HRP)-conjugated secondary antibodies for 1 h at room temperature. The immunoblots were carried out for imaging with the Western blot luminescence detection kit (Santa Cruz Biotechnology, Santa Cruz, CA, USA), and the presences of ZO-1, occludin, NF-кB p65, p38 MAPK, ERK MAPK, and GAPDH were automatically exposed by AlphaImager 2200 (Alpha Innotech, San Leandro, CA, USA) with an appropriate exposure time. Band densities were finally quantified using AlphaImager 2200 as well (Alpha Innotech, San Leandro, CA, USA).

### LDH assay

LDH, a cytoplastic enzyme, will be released into the bloodstream when there is a damage to cells. Therefore, after previously described treatments, the supernatants containing released LDH of cultured IPEC-J2 cells were collected to determine the integrity of cells. IPEC-J2 cells were first harvested and resuspended with LDH assay buffer. After centrifugation at 12,000 r/min for 5 min at 4 °C, supernatants were carefully collected and transferred into the clean tubes. Reaction mix for the assay was prepared by using LDH assay kit (Nanjing Jiancheng Bioengineering Institute, Nanjing, China) according to the manufacturer’s instructions and added into each well with the sample or the control. The fluorescence was immediately measured at 450 nm and the obtained values were analyzed based on the protocol to monitor the activity of LDH. Each sample was assayed for three replicates.

### Analysis of cytokines

IPEC-J2 cells were seeded into 24-well culture plates and used for later experiments until the cell density reached 80% confluence. IL-1β, TNF-α and IL-8 production were measured from the supernatants of IPEC-J2 cells treated or untreated with ETEC K88 (1 × 10^3^ CFU/mL) or *L. salivarius* (1 × 10^5^ CFU/mL) either alone or simultaneously coupled with ETEC K88 for 3 h till 24 h. The cells only treated with *L. salivarius* were set as the control group. Distinct cytokine levels were quantitatively assessed by ELISA using a commercial ELISA kit (Nanjing Jiancheng Biology Engineering Institute, Nanjing, China).

### The functions of MAPK and NF-κB involved in TLRs- and NLRs-triggered signaling pathways during the process of *Lactobacilli* regulating the inflammatory response

SB-203580 (MAPK inhibitor, GlpBio, Montclair, CA, USA), SCH772984 (ERK inhibitor, GlpBio, Montclair, CA, USA) and BAY11–7082 (NF-κB inhibitor, GlpBio, Montclair, CA, USA) were first employed in IPEC-J2 cells for 1 h [19–21], followed by *L. salivarius* involvement for 3 h. After 3 times washes with PBS, IPEC-J2 cells were infected with ETEC K88 for 3, 6, 12 and 24 h, respectively. Non-infected cells were considered as the control group. The cell supernatants were collected from each group, and then the pro-inflammatory cytokines, including IL-1β, TNF-α, IL-8, were detected by ELISA according to the manufacture’s protocols.

### Statistical analysis

All experiments were performed with at least three independent replicates. Statistical analysis was completed using GraphPad Prism 5, and differences were evaluated by Student’s *t*-test. *P*-value of < 0.05 was considered statistically significant.

## Results

### Inhibited adhesion of ETEC K88 to IPEC-J2 cells

**Fig. 1:**
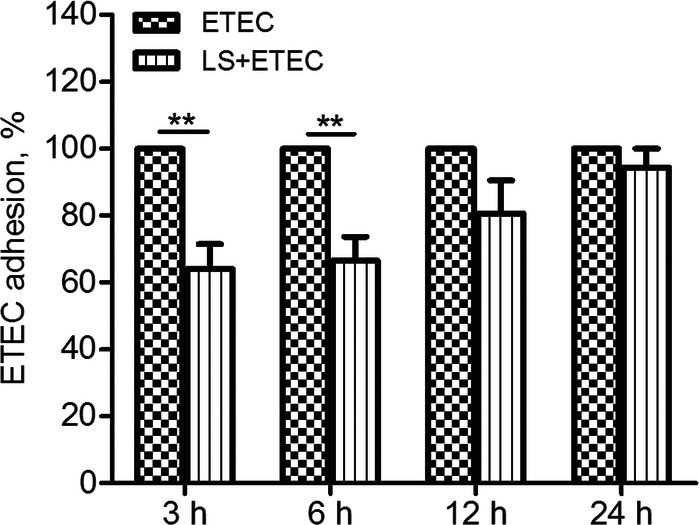
Effects of *L. salivarius* on ETEC K88 adhesion to IPEC-J2. Cells were collected from the indicated IPEC-J2 cultures at 3, 6, 12, 24 h after ETEC K88 challenge. An adhesion assay using ETEC K88 alone served as a reference, and the adherent ratio of ETEC K88 group was normalized to 100%. Data are presented as means ± standard deviations (SD) of three independent experiments. **, *P* < 0.01

The adhesion of ETEC K88 to *L. salivarius*-pretreated IPEC-J2 cells was reduced to varying degrees during the infection. The ratio of the adherent rate of ETEC in cells added with *L. salivarius* to that in the control group was 64% (*P* = 0.0081), 67% (*P* = 0.0091), 80% (*P* = 0.1236), and 94% (*P* = 0.3739), respectively (Fig.1). Internalization of ETEC K88 by IPEC-J2 was not observed.

### Effects of *L. salivarius* on TLRs- and NLRs-mediated signaling pathways

**Fig. 2:**
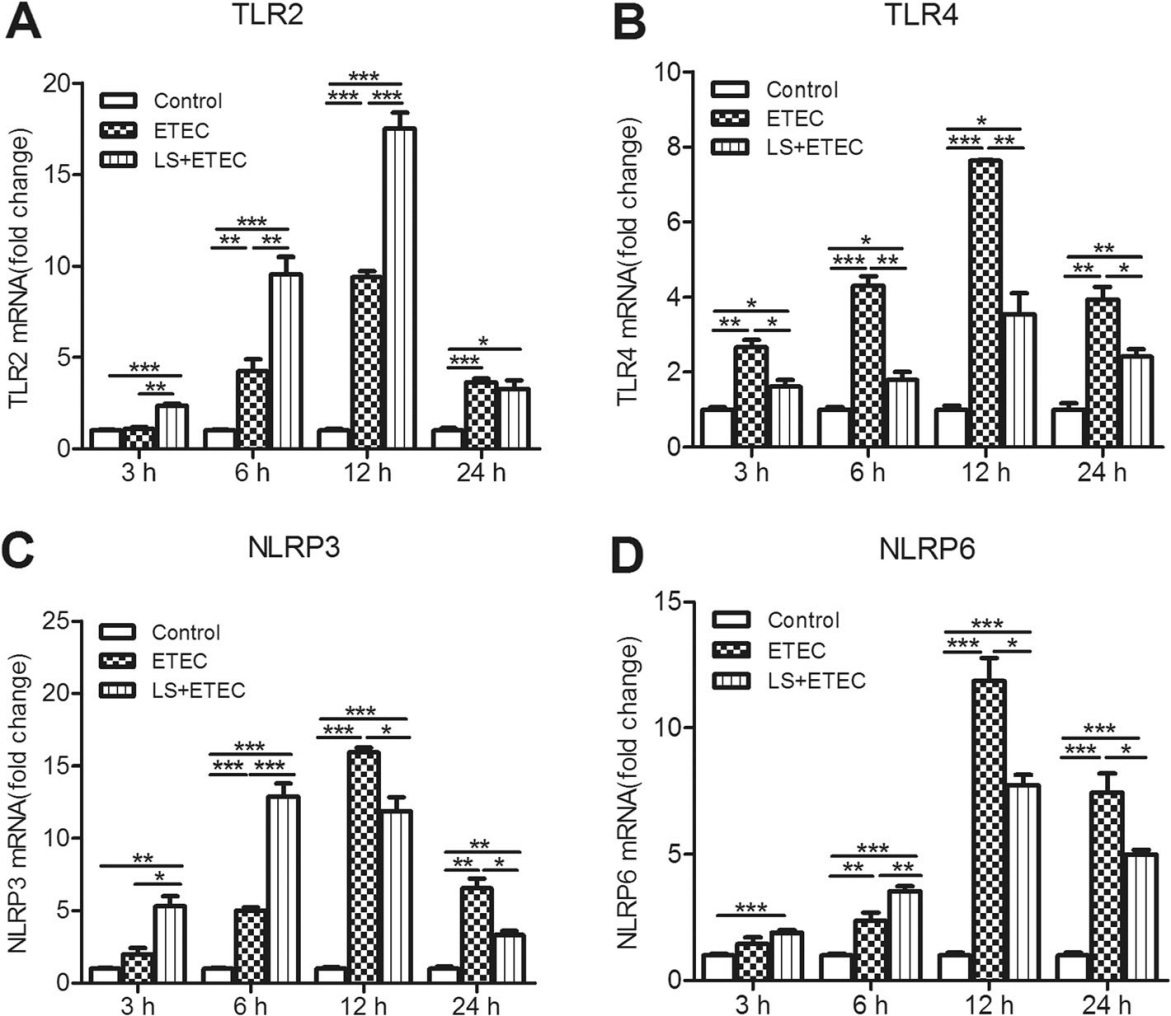
Effects of *L. salivarius* on TLR- and NOD-mediated inflammatory signaling pathways. IPEC-J2 cells collected from the 6-well dishes at 3, 6, 12, 24 h after ETEC K88 challenge. The relative expressions of mRNA for (**a**) TLR2, (**b**) TLR4, (**c**) NLRP3 and (**d**) NLRP6 were analyzed by quantitative real-time PCR. Data are presented as means ± SEM of three independent experiments. *, *P* < 0.05; **, *P* < 0.01; ***, *P* < 0.001

To investigate the effects of *L. salivarius* on the inflammatory process triggered in ETEC K88-challenge IPEC-J2 cells, mRNA levels of TLRs and NLRs were basically detected in three different groups, namely the group of IPEC-J2 cells treated with ETEC K88 alone, the group of cells preincubated with *L. salivarius* before ETEC K88 infection and the control group without any stimulation. After ETEC K88 stimulation for 3, 6 and 12 h, the mRNA level of TLR2 was significantly higher in IPEC-J2 cells with *L. salivarius* supplementation when compared with either the control group or ETEC K88 alone group. (*P* < 0.01, Fig. 2a). However, there was no obvious difference of TLR2 mRNA observed between ETEC K88 alone group and *L. salivarius* pretreated one with a constant challenge from ETEC K88 for 24 h (*P* > 0.05). The qPCR analysis also showed a pronounced down-regulation of TLR4 mRNA in *L. salivarius* pretreated cells compared with ETEC K88 alone group at 3, 6, 12 and 24 h after ETEC K88 challenge (*P* < 0.05 or *P* < 0.01, Fig. 2b). Besides, there was a significant elevation of mRNA levels for both NLRP3 and NLRP6 in cells preincubated with *L. salivarius* and ETEC K88 infectious time for them was 3 h or 6 h (Fig. 2 c, d), but the expression level of NLRP3 started to decline after 6 h infection while that of NLRP6 began to drop after 12 h infection (Fig. 2 c, d).

### Activation of MAPK and NF-κB signaling pathways in ETEC K88-stimulated IPEC-J2 cells

**Fig. 3:**
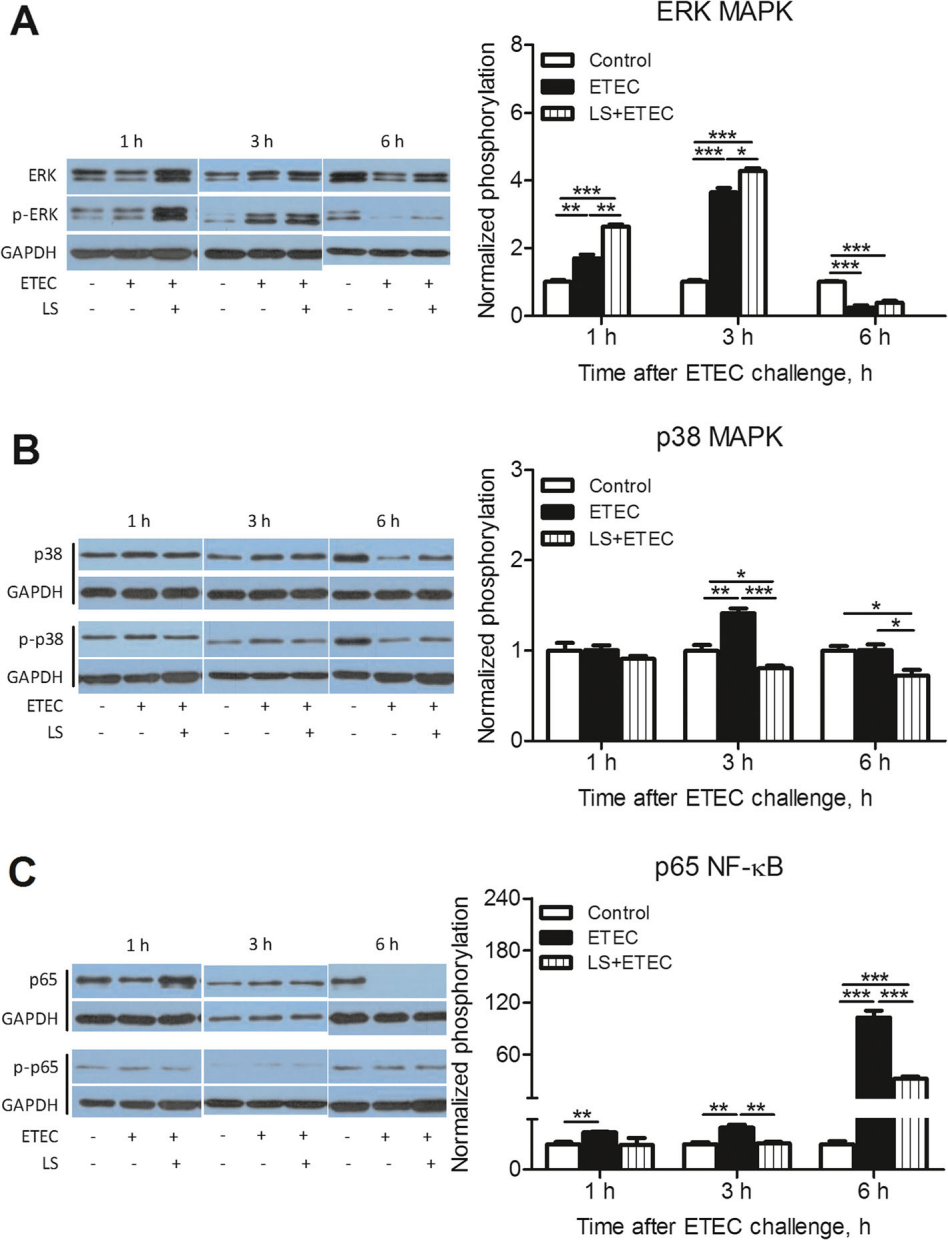
Activation of MAPK and NF-κB pathways in ETEC K88-stimulated IPEC-J2 cells. IPEC-J2 cells harvested from *L. salivarius* involvement groups and control ones were treated with ETEC K88, and cell lysates were subjected to Western blotting with phosphor-specific Abs against (**a**) p-ERK, (**b**) p-p38 and (**c**) p-p65. GAPDH was used as the loading control. Data are presented as means ± SEM of three independent experiments. *, *P* < 0.05; **, *P* < 0.01; ***, *P* < 0.001

To investigate the potential signaling pathways that were responsible for the release of inflammation-related cytokines, the phosphorylation of certain proteins, specifically the MAPK family and NF-κB were examined by Western blotting (Fig. 3). The result indicated that p38 MAPK, ERK MAPK, as well as p65 NF-κB were rapidly phosphorylated with ETEC K88 stimulation in IPEC-J2 cells. Interestingly, the phosphorylation of ERK was markedly increased in *L. salivarius*-pretreated IPEC-J2 cells compared with ETEC K88 alone group after ETEC K88 infection for 1 h (*P* < 0.01) or 3 h (*P* < 0.05) (Fig. 3a). However, *L. salivarius* enabled phosphorylation of p38 and p65 to substantially decline in cells infected with ETEC K88 for either 3 h or 6 h when compared with ETEC K88 alone group (*P* < 0.05 or *P* < 0.01; Fig. 3b, c).

### The cytokine alterations with *Lactobacillus* pretreatment

**Fig. 4:**
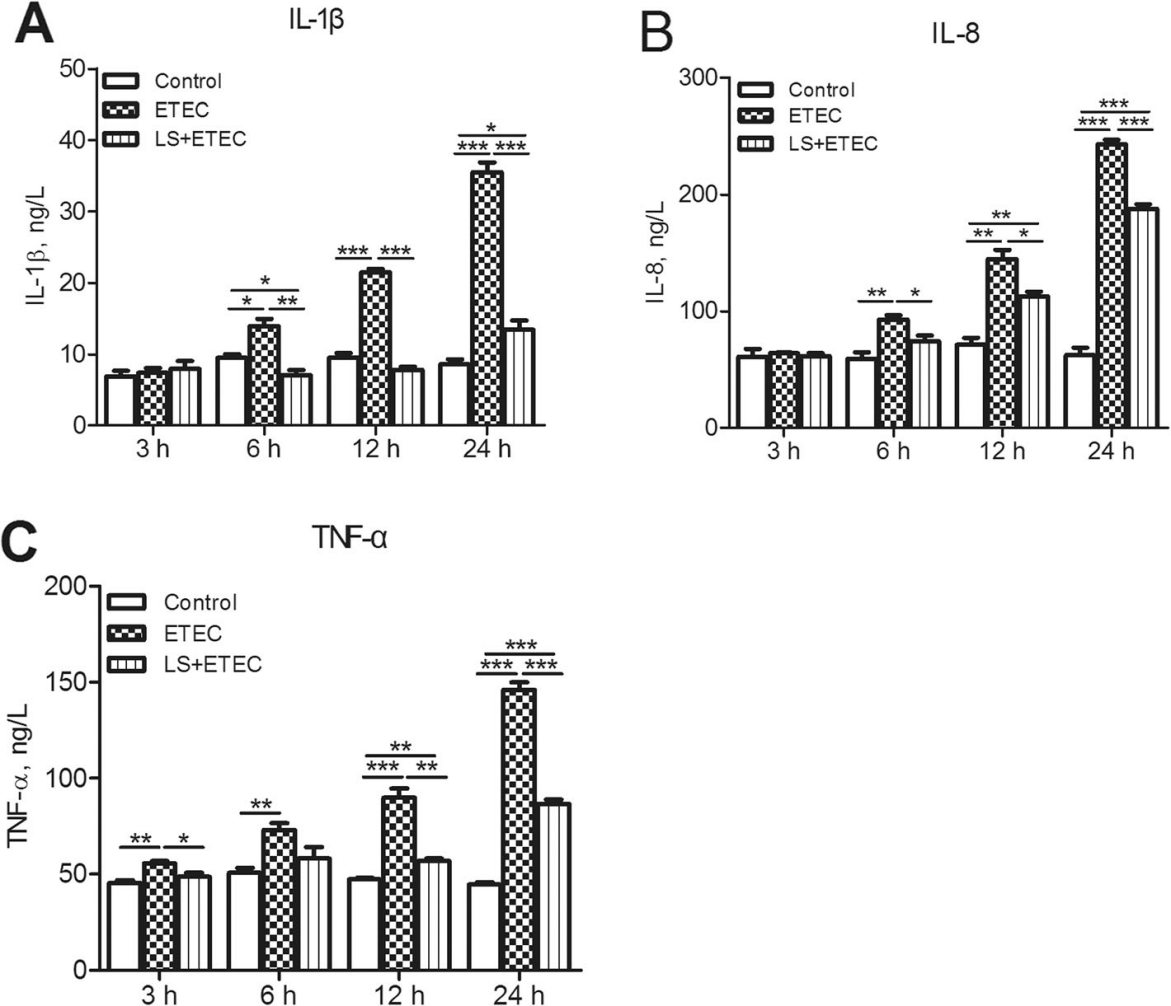
Effects of *L. salivarius* on cytokine release in IPEC-J2 cells challenged with ETEC K88. The supernatant collected from the indicated IPEC-J2 cultures at 3, 6, 12, and 24 h after ETEC K88 challenge and the release of (**a**) IL-β, (**b**) IL-8 and (**c**) TNF-α were analyzed by ELLSA. Data are presented as means ± SEM of three independent experiments. *, *P* < 0.05; **, *P* < 0.01; ***, *P* < 0.001

Inflammatory status of IPEC-J2 cells was evaluated by measuring the expression levels of pro-inflammatory cytokines (IL-1β, IL-8 and TNF-α). ETEC K88 treatment for 6 h till 24 h resulted in the increased levels of IL-1β and IL-8 (*P* < 0.05 or *P* < 0.01, Fig. 4 a-b) whereas 3 h till 24 h infection induced a significant augmentation of TNF-α (Fig. 4 c). Meanwhile, the assistance of *L. salivarius* obviously decreased the expression levels of the above three induced cytokines (Fig. 4 a-c). It is worth noticing that there were no significant distinctions of IL-1β, IL-8, and TNF-α levels between the *L. salivarius*-pretreated cells and the control ones when ETEC K88 challenging time was 3 h. (*P* > 0.05).

### Positive influence of *L. salivarius* on expression levels of tight junction proteins

**Fig. 5:**
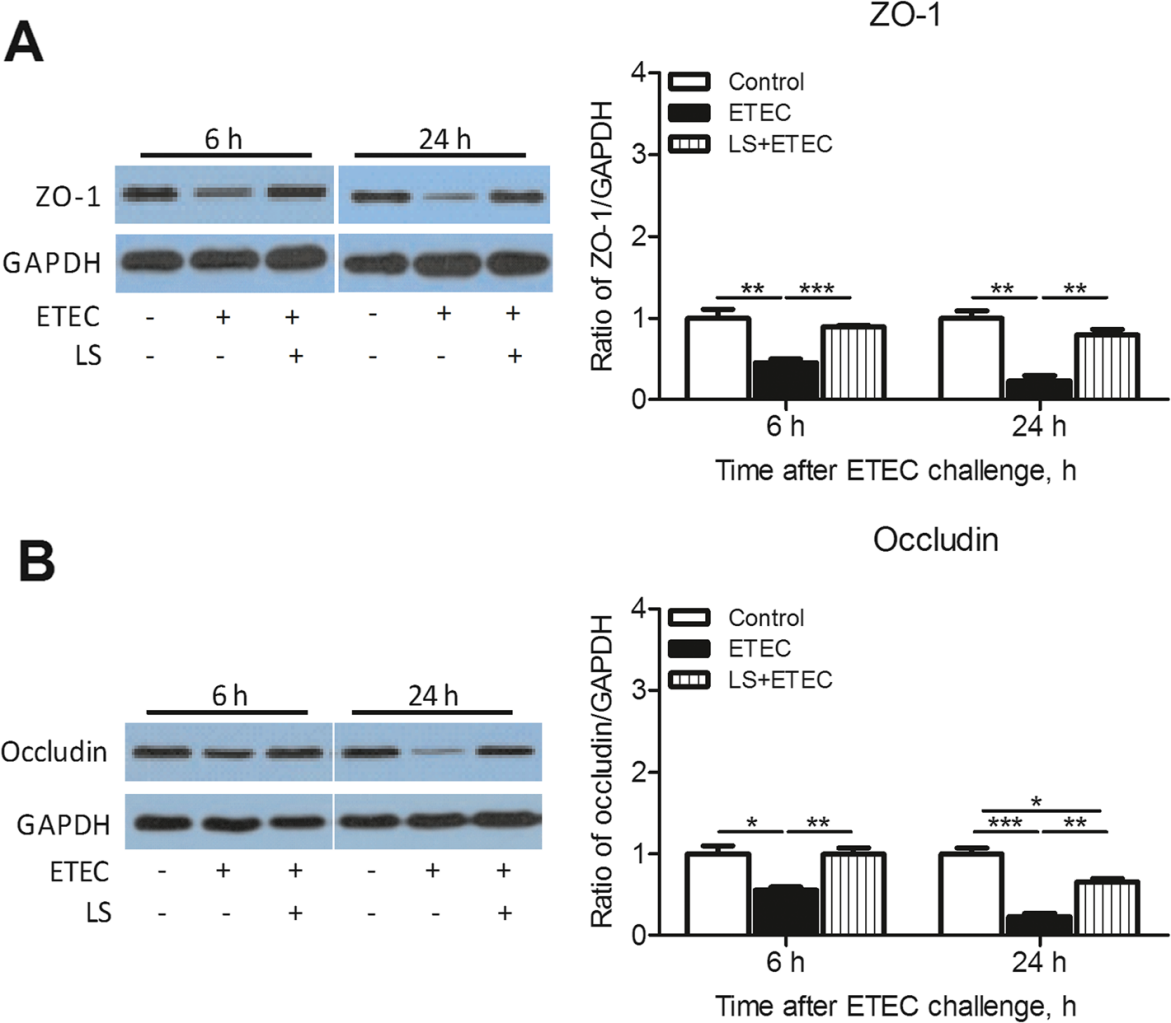
Augmentation of tight junction proteins in IPEC-J2 cells treated with *L. salivarius* at 6 and 24 h after ETEC K88 challenge. (**a**) ZO-1 and (**b**) occludin were detected by Western blotting. GAPDH were used as the loading control. Data are presented as means ± SEM of three independent experiments. *, *P* < 0.05; **, *P* < 0.01; ***, *P* < 0.001

To investigate the possible membrane barrier disruption caused by ETEC K88 and the potential role of *L. salivarius* in restoring such damage, an immunobolt of ZO-1 and occludin was performed (Fig. 5). ETEC K88 was able to obviously decrease expression levels of ZO-1 and occludin in IPEC-J2 cells (*P* < 0.05) while *L. salivarius* involvement prior to infection had the capability to significantly increase the levels of these two tight junction proteins (*P* < 0.05, Fig. 5).

### Effects of *L. salivarius* on LDH production

**Fig. 6:**
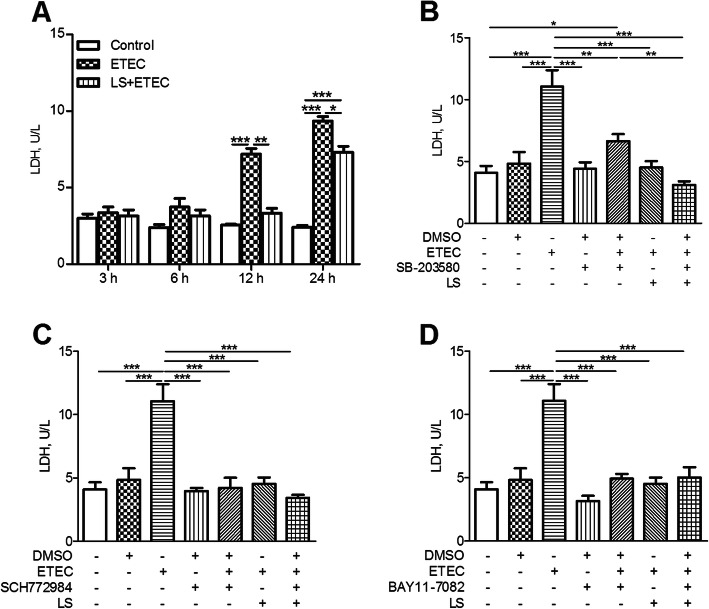
Concentration of LDH in supernatants of IPEC-J2 cells. IPEC-J2 cells added with DMSO were regarded as the vehicle control and *L. salivarius*-involved IPEC-J2 cells were supplemented (**a**) without inhibitor, (**b**) with 10 μmol/L SB-203580 (p38 MAPK inhibitor), (**c**) with10 μmol/L SCH772984 (ERK MAPK inhibitor) and (**d**) with 10 μmol/L BAY11-7082 (NF-κB inhibitor), respectively. Concentration of LDH was detected after ETEC K88 challenge. Data are presented as means ± SEM of three independent experiments. *, *P* < 0.05; **, *P* < 0.01; ***, *P* < 0.001

To identify whether ETEC K88 caused cell membrane damage, LDH in IPEC-J2 cell culture medium was detected. Challenge with ETEC K88 for 12 and 24 h significantly increased LDH content in IPEC-J2 cells (*P* < 0.05, Fig. 6a), indicating an increased cell permeability. As expected, *L. salivarius* treatment reduced LDH released from the cells (Fig. 6a). In addition, *L. salivarius* along with either MAPK inhibitors or NF-κB inhibitors brought an obviously reduced level of LDH concentration in the medium. (*P* < 0.05, Fig.6 b-d).

### The functions of MAPK and NF-κB involved in TLRs- and NLRs-triggered signaling pathways during the process of *Lactobacilli* regulating the inflammatory response

**Fig. 7:**
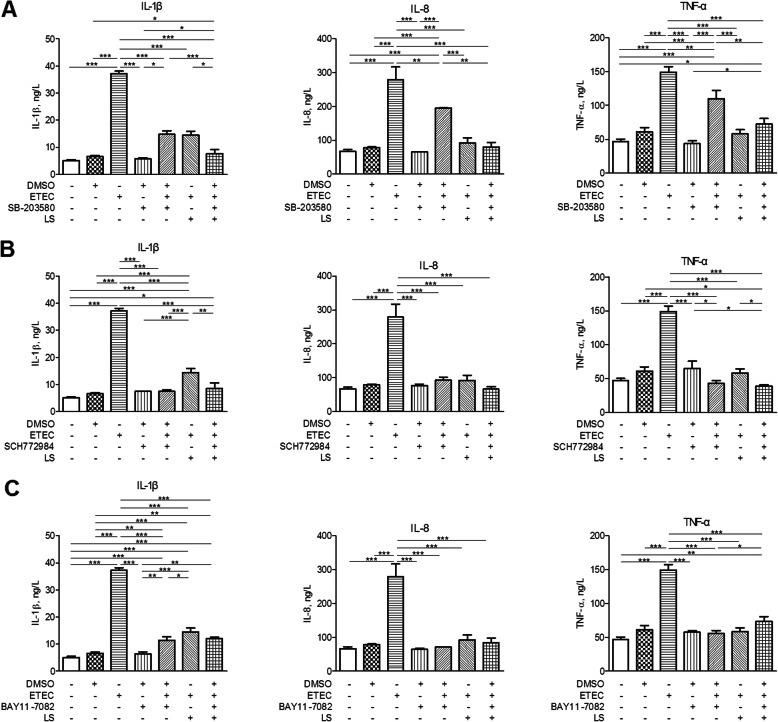
Concentrations of IL-8, IL-1β and TNF-α in supernatants of IPEC-J2 cells. IPEC-J2 cells added with DMSO were regarded as the vehicle control and *L. salivarius*-involved IPEC-J2 cells were supplemented with (**a**) 10 μmol/L SB-203580, (**b**) 10 μmol/L SCH772984 and (**c**) 10 μmol/L BAY11-7082, respectively. Concentrations of TNF-α, IL-8 and IL-1β were detected after ETEC K88 challenge. Data are presented as means ± SEM of three independent experiments. *, *P* < 0.05; **, *P* < 0.01; ***, *P* < 0.001

Since phosphorylation of p38 MAPK was attenuated in ETEC K88-stimulated IPEC-J2 cells in response to *L. salivarius*, the potential effect of specific MAPK inhibitors on levels of IL-1β, IL-8, and TNF-α production were further examined. IPEC-J2 cells were added with SB-203580 (p38 MAPK inhibitor) and SCH772984 (ERK MAPK inhibitor), respectively for 1 h and then stimulated with ETEC K88 for 24 h. It turned out that the existence of either SB-203580 or SCH772984 could induce the declined levels of IL-1β, IL-8, and TNF-α in groups with *L. salivarius* supplementation and the control ones (Fig. 7a, b). BAY11–7082 (NF-κB inhibitor) was also applied for 1 h before ETEC K88 stimulation for 24 h, aiming to present the potential role of NF-κB pathway in the process of *L. salivarius* affecting the production of IL-1β, IL-8, and TNF-α. However, the elevating levels of pro-inflammatory cytokines were detected in ETEC K88 infected groups with the supply of *L. salivarius* (Fig. 7c)*.*

## Discussion

The application of probiotics has been proved to bring a vast array of well-established effects, including the regulation of pro- and anti-inflammatory cytokines, the enhancement of secretory IgA, the production of antibacterial substances, the preservation of intestinal barrier integrity, and the competition with pathogenic microorganisms for enterocyte binding. One previous study indicated that *Lactobacillus* might have a protective effect on the intestinal cells against the inflammatory status and mucosal injury triggered by ETEC K88 infection [22]. However, the basic molecular mechanism by which *Lactobacillus* might contribute to the regulation of intestinal epithelial cells still remains ambiguous and requires follow-up exploration.

The ability of the bacterial adhesion to host epithelial cells is regarded as one of the key steps for pathogenesis, which encourages some researches to focus on the adherent capability of ETEC K88 to IPEC-J2 cells with *L. salivarius* preincubation. One of our results demonstrated that *L. salivarius* administration made the adherent rate of ETEC K88 to IPEC-J2 cells drop 36%, 33%, 20%, 6% at 3, 6, 12, and 24 h after infection, respectively (Fig. 1). The proteins anchored on the probiotic surface layer as well as some extracellular polysaccharides may prevent the formation of pathogenic biofilms by inhibiting adherence and blocking the binding sites of pathogenic bacteria to intestinal cell surface receptors. The adhesion of probiotic bacteria to epithelial cells also appear to be a contributing factor for optimizing bacterial colony balance in the pig intestine [23, 24]. Taken together, since *L. salivarius* could be able to effectively inhibit ETEC K88 adherence to porcine intestinal epithelial cells, this probiotic might have a defensive role in eliminating infectious process and improving the health of piglets.

Intestinal barrier is believed to be closely associated with functional tight junctions which are defined as dynamic complex locating between epithelial cells and function as a critical structure for paracellular permeability based on molecular charge and size [25]. In this sense, any alternation of this multi-functional and continuous assemble may contribute to the limitation of paracellular permeability and make entry or net flow of ions and solutes possible. Tight junctions comprise of transmembrane proteins (claudin and occludin) as well as various cytosolic proteins that are recruited to the apicolateral membrane, including zonula occludin protein 1 (ZO-1), ZO-2, ZO-3, cingulin, and 7H6 [26]. The integrity and regular permeability of tight junctions were negatively influenced in IPEC-J2 cells with invasion of ETEC K88 [27]. Plus, ETEC K88 infection could increase the permeability of tight junctions in early weaned piglets [28] and *L. reuteri* LR1 was proved to reduce ETEC K88-induced membrane barrier disruption by maintaining proper position of ZO-1 [29]. These supported evidences were consistent with our presented data that ETEC K88 challenge could decrease the expression of ZO-1 and occludin in IPEC-J2 cells, and these down-regulations could be partially reversed by the application of *L. salivarius* prior to bacterial infection (Fig. 5).

LDH is an oxidoreductase enzyme found in the cytoplasm and is widespread in tissues. Previous knowledge showed that both intracellular or extracellular stress resulted in an impaired mucosal barrier function and stimulated subsequent release of LDH from cells (as indicated by the amount of LDH in the culture medium) [30]. Thus, the amount of LDH detected in the culture medium is generally used as an indicator for determining damaged or dead cells. In our study, we found that ETEC K88 was able to induce inflammatory reaction in IPEC-J2 cells and highly augment LDH release from these cells. It’s noteworthy to notice that the addition of *L. salivarius* to the culture medium markedly decreased LDH release from ETEC K88-induced cells (Fig. 6), which presented an involved role of *L. salivarius* in protecting the integrity of IPEC-J2 cell membrane.

The innate immune response is initiated by cell surface PRRs recognizing invading microorganisms and then down-streaming signals will be consequently activated to produce a variety of biological effects. TLRs and NLRs are two important PRRs and the roles of which in the immune system have been well established [31]. In the present study, ETEC K88 appeared to be a causing factor for the significant increase of TLR2 mRNA expression and the pretreatment of *L. salivarius* in ETEC K88-infected groups further elevated the mRNA expression of TLR2 within 3–12 h infection (Fig. 2a). *L. paracasei* was reported with an ability to inhibit proinflammatory cytokines by monocyte-macrophages via the induction of negative regulators for TLR2-dependent activation of NF-κB signaling pathways [32]. The decreased phosphorylation of p65 NF-κB caused by *L. salivarius* might be related to the activation of TLR2. Thus, we hypothesize that activation of *L. salivarius*-mediated TLR2-dependent signaling pathways might induce the recruitment and activation of some inflammatory cells, serving as a protective and defensive strategy for host cells to resist ETEC K88-induced inflammation. Consistent with our results, the expression level of TLR2 in cells treated with either *L. rhamnosus* GR-1 [33] or *L. plantarum* NDC 75017 [34] increased as well. However, an inhibitory impact of *L. salivarius* on TLR4 expression was shown in infectious IPEC-J2 cells, which was opposite to the change of TLR2 expression in the same group. Due to the fact that TLR4 is the main receptor for recognizing LPS, a vital component of *Escherichia* cell wall [35], it is no wonder that level of TLR4 in ETEC K88-infected groups was relatively higher than that in the control groups in this study (Fig. 2b).

NLRs are cytoplasmic counterparts to TLRs and function in initiating the innate immune response with the appearance of pathogens [36]. NLRP3 and NLRP6 expression were both higher in groups with pathogenic involvement for 3–6 h, but this pattern started to decrease with an extended ETEC infectious time period for 12–24 h (Fig. 2 c, d). It is well appreciated that TLRs and NLRs are able to recruit adaptor protein MyD88 to activate a cascade of downstream signal transduction. For instance, MAPK and NF-κB are common targets for activation and mainly responsible for regulating related gene expression [15, 37]. A large number of investigators have confirmed the above statements and one study reported that the decreased expressions of TLRs and NLRs caused the attenuated phosphorylation of MAPK and NF-κB, resulting in a decreased secretion of diverse cytokines [38]. Our data suggest that *L. salivarius* enabled the expression levels of TLR4, NLRP3 and NLRP6 to reduce in cells infected with ETEC K88. Previous data demonstrated the protective role of probiotics *L.amylovorus* and *L. jensenii* in down-regulating TLR4-dependent NF-κB and MAPK activation as well as triggering negative regulators of TLRs, including Tollip, Bcl-3, A20, MKP-1, and inhibitory IL-1R-related kinases [39, 40]. Their findings coupled with ours provide a possibility of probiotics to protect intestinal cells from ETEC K88 injury by alleviating inflammatory response. In addition, ERK1/2 and p38 are two distinct groups of MAPKs and involved in the inflammatory response. In this study, *L. salivarius* stimulation caused an inhibited phosphorylation of p38 MAPK in cells infected with ETEC for 1 or 3 h. However, an enhancement of ERK phosphorylation was observed in the same group (Fig. 3a, b).

NF-κB, a transcription factor, is known as an important regulator for controlling the generation of multiple inflammation-related cytokines like TNF-α. The production of TNF-α and the activation of NF-κB form a positive feedback loop in which the activation of NF-κB is followed by the production of TNF-α which further initiates signaling cascades and activates NF-κB [41]. Protein phosphorylation is central to the regulation of the signal transduction systems since it acts as a switch to turn protein activity on or off. Therefore, the phosphorylation of NF-κB subunit was measured in this research, and the result revealed the p65 subunit of NF-κB phosphorylation was significantly inhibited with *L. salivarius* treatment (Fig. 3c). Therefore, we speculate that *L. salivarius* might exert an anti-inflammatory effect in ETEC K88-induced IPEC-J2 cells at least in part via the NF-κB and MAPK-dependent signaling pathways.

Cytokine production is considered as an important indicator in response to ETEC K88. Even though some studies have demonstrated that LAB strains could trigger intestinal epithelial cells to produce pro-inflammatory cytokines [42, 43], we found that *L. salivarius* pretreatment prior to pathogenic exposure actually attenuated the release of cytokines IL-1β, IL-8 and TNF-α (Fig. 4). In line with our findings, *L. plantarum* could suppress proinflammatory cytokine production by inhibiting both NF-κB and p38 MAPK [44]. Other studies also demonstrated that the expression of pro-inflammatory cytokines like IL-8 and IL-6 would be down-regulated by *L. reuteri* [29]. These studies were confirmed by using the application of specific signaling inhibitors, which decreased cytokine levels in ETEC K88-induced cells (Fig. 7a, b, c). These results suggest that *L. salivarius* could regulate cytokines, probably slowing down cell damage and relatively weakening inflammation. In conclusion, our data suggest that *L. salivarius* might inhibit IL-1β, IL-8, and TNF-α production due to the inhibition of NF-κB signaling pathway or the attenuated phosphorylation of p38 MAPK. It might also be possible for *L. salivarius* to enhance the cell integrity according to the up-regulated expression of ZO-1 and occludin in tight junctions.

## Conclusions

This study may provide useful information for the development of potential therapeutic strategies for the prevention or improvement of intestinal diseases in piglets using *L. salivarius*. At the same time, our data partly reveal the underlying mechanism of *L. salivarius* benefiting piglet health and provide a theoretical basis for the application of this probiotics in piglet diets.

## Data Availability

All data generated or analyzed during this study are available from the corresponding author by request.

## Abbreviations

CFU: Colony forming unit
ERK: Extracellular signal-regulated kinase
ETEC: Enterotoxigenic *Escherichia coli*
h: Hour
IL: Interleukin
IPEC-J2: Intestinal porcine epithelial cell line
LB: Luria-Bertani
LDH: Lactate dehydrogenase
*L. salivarius*: *Lactobacillus salivarius*
MAPK: Mitogen-activated protein kinase
min: Minute
MRS: De Man, Rogosa and Sharpe
NF-κB: Nuclear factor-κB
NLRP: NOD-like receptor pyrin domain-containing protein
NLRs: NOD-like receptors
NOD: Nucleotide-binding oligomerization domain
PAMPs: Pathogen-associated molecular patterns
pBD-2: Porcine β-defensin-2
PBS: Phosphate buffer saline
PCR: Polymerase chain reaction
PRRs: Pattern recognition receptors
PVDF: Polyvinylidene difluoride
RPMI: Roswell park memorial institute
TLR: Toll like receptor
TNF-α: Tumor necrosis factor-α
ZO-1: Zona occludens 1
